# Small-scale dosimetry for alpha particle ^241^Am source cell irradiation and estimation of γ-H2AX foci distribution in prostate cancer cell line PC3

**DOI:** 10.1186/s40658-022-00475-x

**Published:** 2022-07-19

**Authors:** Emma Mellhammar, Magnus Dahlbom, Oskar Vilhelmsson-Timmermand, Sven-Erik Strand

**Affiliations:** 1 Department of Clinical Sciences Lund, Oncology, Lund University, Barngatan 4, 221 85 Lund, Sweden; 2 Department of Molecular and Medical Pharmacology, David Geffen School of Medicine at UCLA, Los Angeles, CA USA; 3 Imaging Chemistry and Biology, Kings Collage London, London, UK; 4 Department of Clinical Sciences Lund, Medical Radiation Physics, Lund University, Lund, Sweden

**Keywords:** Small-scale dosimetry, Alpha particles, Monte Carlo simulation, γ-H2AX, PC3

## Abstract

**Background:**

The development of new targeted alpha therapies motivates improving alpha particle dosimetry. For alpha particles, microscopic targets must be considered to estimate dosimetric quantities that can predict the biological response. As double-strand breaks (DSB) on DNA are the main cause of cell death by ionizing radiation, cell nuclei are relevant volumes necessary to consider as targets. Since a large variance is expected of alpha particle hits in individual cell nuclei irradiated by an uncollimated alpha-emitting source, the damage induced should have a similar distribution. The induction of DSB can be measured by immunofluorescent γ-H2AX staining. The cell γ-H2AX foci distribution and alpha particle hits distribution should be comparable and thereby verify the necessity to consider the relevant dosimetric volumes.

**Methods:**

A Monte Carlo simulation model of an ^241^Am source alpha particle irradiation setup was combined with two versions of realistic cell nuclei phantoms. These were generated from DAPI-stained PC3 cells imaged with fluorescent microscopy, one consisting of elliptical cylinders and the other of segmented mesh volumes. PC3 cells were irradiated with the ^241^Am source for 4, 8 and 12 min, and after 30 min fixated and stained with immunofluorescent γ-H2AX marker. The detected radiation-induced foci (RIF) were compared to simulated RIF.

**Results:**

The mesh volume phantom detected a higher mean of alpha particle hits and energy imparted (MeV) per cell nuclei than the elliptical cylinder phantom, but the mean specific energy (Gy) was very similar. The mesh volume phantom detected a slightly larger variance between individual cells, stemming from the more extreme and less continuous distribution of cell nuclei sizes represented in this phantom. The simulated RIF distribution from both phantoms was in good agreement with the detected RIF, although the detected distribution had a zero-inflated shape not seen in the simulated distributions. An estimate of undetected foci was used to correct the detected RIF distribution and improved the agreement with the simulations.

**Conclusion:**

Two methods to generate cell nuclei phantoms for Monte Carlo dosimetry simulations were tested and generated similar results. The simulated and detected RIF distributions from alpha particle-irradiated PC3 cells were in good agreement, proposing the necessity to consider microscopic targets in alpha particle dosimetry.

**Supplementary Information:**

The online version contains supplementary material available at 10.1186/s40658-022-00475-x.

## Background

Alpha-emitting radionuclides have long been candidates for radionuclide therapy (RNT) to treat cancer, with promising attributes needed to achieve high local absorbed doses. Due to the short range of the emitted alpha particles, sparing nontargeted tissues while eradicating tumor cells is theoretically possible. The high linear energy transfer (LET) of alpha particles generates complex damage of multiple DNA double-strand breaks (DSB) in the cell nucleus that are, if not impossible, at least very challenging for cellular DNA damage repair mechanisms to remedy [1–3].

Radium-223 is the first alpha-emitting radionuclide approved for RNT for cancer. Radium-223 dichloride is used clinically for palliative treatment of metastatic castration-resistant prostate cancer with bone metastases. As a calcium analog, it is taken up in regions of osteoblastic activity, such as bone metastasis [4]. Radium-223 has been shown to accumulate in the bone microenvironment close to the tumors and reduces abnormal bone growth, reducing pain, and slightly improving overall survival [5].

In recent years, several trials of targeted alpha therapy (TAT) have shown promising results, both concerning safety and efficacy [2]. Clinical trials treating metastatic castration-resistant prostate cancer with ^225^Ac-PSMA-617 have shown a clear antitumor activity in advanced-stage patients [6, 7]. A recent meta-analysis of ^225^Ac-PSMA-TAT data found a PSA decline greater than 50% was seen in 83% of patients and complete molecular response in 17% of patients [8]. Except for ^223^Ra and ^225^Ac previously mentioned, other feasible radionuclide candidates for TAT include ^227^Th, ^213^Bi, ^211^At, ^212^Pb and ^149^ Tb [2, 9].

As the efforts to utilize alpha-emitting radionuclides for TAT are intensifying, the need to comprehend the underlying mechanics of the damage induction of short-range, high-energy alpha particles in tissue is urgent. The basics of alpha radiobiology rest heavily on in vitro experiments, and the accompanying dosimetry models need to be accurate to be effective in predicting biological outcome. To accomplish this, the radiation interactions on a subcellular level are critical to model, as singular alpha particle tracks can induce cell death.

In vitro cell irradiations with alpha particles are often performed with either a collimated microbeam, the addition of an alpha-emitting radiopharmaceutical to the target cells culture growth media or with an uncollimated source exposing a surface with adherent cells. In the latter two cases, there is a large variance in the number of alpha particle tracks that irradiated cells will be exposed to. Consequently, the absorbed dose, or in microdosimetric terms, the specific energy [10], in the cells or cell nuclei has a high variability [11]. Similarly, intratumoral variation of dosimetric quantities is to be expected in tumors with uptake heterogeneities for radiopharmaceuticals emitting radiation of short range [12]. In this aspect, uncollimated source irradiations to some degree mimic the complex in vivo dosimetric case, but is less complex to perform in comparison with the addition of a radiopharmaceutical to the culture media, where the uptake and internalization need to be quantified to build a robust dosimetry model.

At low absorbed doses of alpha particles (approx. 0–1 Gy), a substantial portion of the cells might not have a single track passing through the cell nucleus [13]. The cells that are hit experience a high absorbed dose, higher than what is estimated by the macroscopic absorbed dose, since the energy imparted in individual cell volumes is then divided by all cells exposed, including cell volumes never actually hit.

Commonly, conventional cell irradiation dosimetry consists of calculating the absorbed dose in a water volume many times bigger than the cells or cell nuclei. This is a major drawback when used for short-range radiation and can lead to misestimation of the surviving fraction of an irradiated cell population or incorrect calculation of the tumor control probability of a targeted tumor. Indeed, as defined by ICRU Report 36, the specific energy is a stochastic quantity, while absorbed dose is deterministic [10].

Histone Ser-139 γ-H2AX, a marker for DNA DSBs, has become a possible tool for in vitro and in vivo dosimetry, including irradiations with alpha emitters [14, 15]. After the induction of a DSB, phosphorylation of histone Ser-139 happens within minutes as a link in the cells damage response signaling chain. Foci at the damage sites can be visualized with fluorescent ser139-specific γ-H2AX antibodies [16].

However, when measuring the number of γ-H2AX foci in an irradiated cell population, the mean number of foci per cell will not reflect the large variation in DNA damage the cells will have experienced due to the variation in number of alpha tracks hitting each cell. This variability should therefore be reflected in the damage distribution as measured by the foci distribution in the irradiated cell population.

By modeling the source and target volumes, i.e., the cell nuclei, and performing Monte Carlo simulations, we can calculate the alpha particle target hit rate. When the source surface is smaller than the target surface, as is true for the irradiation setup used in this work, the radial alpha particle exposure of the target surface is non-uniform, and the uniformity further decreases as the source-to-target distance decreases [17, 18]. If the source and target surfaces are circles centered above one another, then the radiation fluence will decrease as a function of distance from the center. Consequently, cells in the center of the target surface will experience more [19] hits than cells on the surface periphery. With a model describing the source-to-target relationship, this variance can be accounted for.

With this model, we can estimate the relationship between the source and the specific energy distribution. This could then be related to the damage distribution. Microscopic dose simulations in cells have been performed by multiple groups with numerous irradiation setups [20–25].

In this study, we aim to simulate the distribution of damage induced after in vitro alpha irradiation, as measured by γ-H2AX fluorescent immunostaining. The distribution of simulated alpha particle hits in the irradiated cell nuclei will be compared to the detected γ-H2AX foci distribution in irradiated PC3 cells.

## Methods

### Alpha source count rate and energy spectrum

Cell irradiations were performed with a ^241^Am source (Eckert & Ziegler Isotope Products GmbH, Braunschweig, Germany). The source is sealed in an aluminum cover, and the circular active source window is 11.8 mm in diameter and covered by a 0.5 µm palladium coating. The activity was previously estimated to 405 ± 10 kBq [26].

A Passivated Implanted Planar Silicon (PIPS) detector A300 (Canberra, Mirion Technologies) in vacuum was used to measure the emission rate and energy spectrum of alpha particles from the ^241^Am source. All alpha particles reaching the detector are recorded as the detector thickness is greater than the alpha range for the alpha energies investigated in this work. Therefore, the detector efficiency is given by the geometrical efficiency. The source was positioned in the detector vacuum chamber at a 36 mm distance between the source window surface and detector surface.

### Cell culture

The prostate cancer cell line PC3 purchased from the American Type Culture Collection (ATCC®, Manassas, VA, USA) were grown in RPMI-1640 medium with L-glutamine (Biowest, Nuaillé, France) supplemented with 10% fetal bovine serum (Fisher Scientific, UK) and 1% penicillin–streptomycin (Fisher Scientific, UK). Cells were grown in T-25 flasks as a monolayer in a humified atmosphere in an incubator at 5% CO_2_ and 37 °C. Twenty thousand cells were seeded directly on glass coverslips placed at the bottom of wells on a 24-well plate one day prior to alpha irradiation, fixation, and fluorescent staining.

### Alpha and X-ray irradiation

**Fig. 1:**
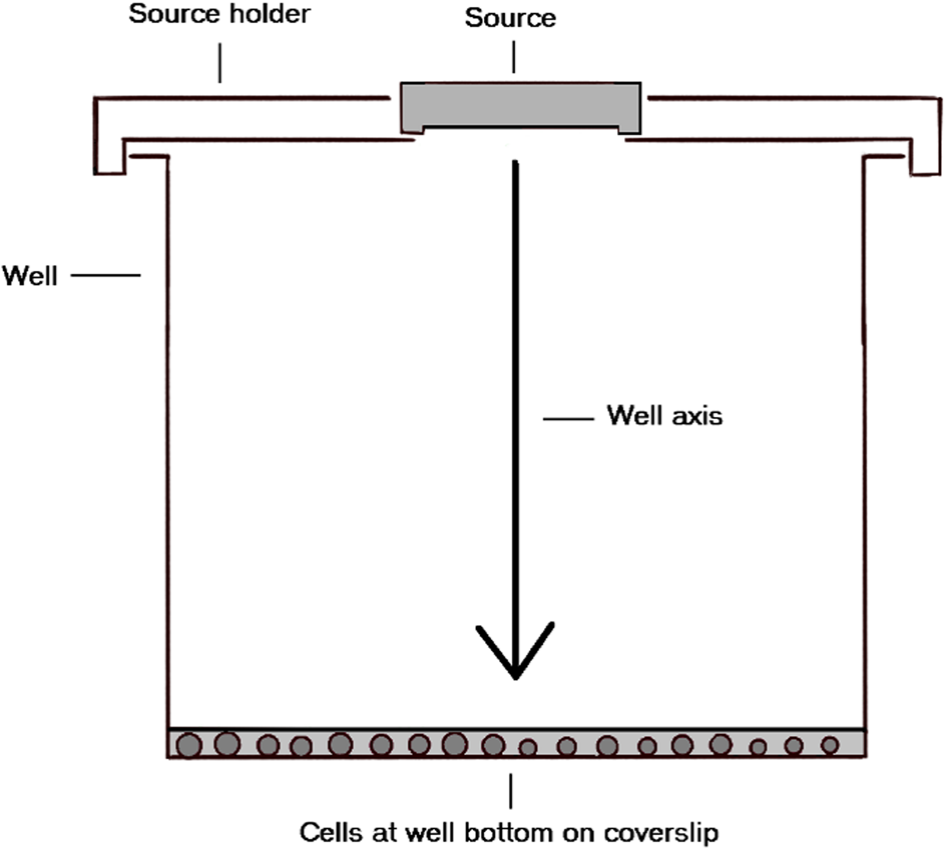
Illustration of alpha irradiation of cells at bottom of well (not to scale). Arrow indicating the direction of the well axis, as referred to in the text. The ^241^Am source is placed in a holder centered over the well opening, irradiating adherent PC3 cells on the bottom. Irradiations are performed with cell media removed. The source-to-bottom distance is 19 mm, and the well is a cylinder of diameter 16.2 mm

When irradiating the PC3 cells with alpha particles, the cell culture media was removed to allow alpha particles to reach the cells (the alpha particle range in water is less than < 50 µm for the relevant particle energies) and the ^241^Am source was placed in a holder on top of the well plate with the source surface centered over the well opening. The distance between source window and the coverslips at the well bottom was 19 mm (Fig. 1). Cells were irradiated for 4, 8, and 12 min in triplicates. The ^241^Am source was immediately removed at the end of the irradiation, and fresh cell culture media was added to the well. To evaluate the damage caused by cells being without culture medium, cells were sham-irradiated for 12 min. The removal of cell media could potentially not only affect the background distribution of foci, but also affect the induction of γ-H2AX RIF and their repair, as compared to an irradiation setup in which the cells are continuously covered by cell media. This is a factor necessary to consider when evaluating the results, but it is beyond the aim of this study to further investigate this.

To compare the appearance of γ-H2AX foci in control, alpha-, and X-ray-irradiated PC3 cells, cells plated on cover slips, as described above, were irradiated to 0.5 Gy on a XneX system (Xstrahl) with a 220 kV tube voltage at a dose rate of 2.1 Gy/minutes.

### Immunofluorescence staining and imaging

Thirty minutes after irradiation, cells were fixated with 4% paraformaldehyde in PBS for 15 min, washed with PBS, permeabilized in 0.1% Triton X-100 solution (Sigma-Aldrich) for 5 min twice, washed with PBS in between and after. Cells were incubated with 1% bovine serum albumin (Sigma-Aldrich) for 30 min. Cells were then incubated with primary antibody (Anti-gamma H2A.X (phosphor S139) antibody [9F3] ab26350) (AbCam) for 1 h and subsequently rinsed with 0.05% Triton X-100 for 5 min twice with PBS rinse in between and after. Then, secondary antibody (Alexa Fluor® 647-conjugated AffiniPure F(ab')2 Fragment Donkey Anti-Mouse IgG) (Jackson ImmunoResearch) incubation lasted 30 min. Cells were rinsed twice in 0.05% Triton X-100 for 5 min. The cells were stained with DAPI (Thermo Scientific) for 15 min and then rinsed twice before mounting the coverslips on slides in antifade solution (Fluoroshield Abcam, ab104135).

Stained cells were imaged on a laser scanning confocal microscope (LSM 710 Confocal Microscope, Zeiss) with 63 × oil immersion objective. The pixel size in the horizontal plane (x and y) was 0.1318 µm and 0.3756 µm in the z-direction. To sample a large surface on the center of the coverslip area, tiled 16-bit images in a 3 × 3 grid were collected so that the resulting images had 3072 × 3072 pixels. Each grid had the length and width of 405 µm. At least 10 tiled grid images were collected for each coverslip. To generate a 3D mesh phantom, a smaller sample of cells on the non-irradiated controls were imaged as a z-stack. When imaging γ-H2AX foci, images were taken in the central 4*4 mm^2^ square of the cover slip.

### Image foci segmentation

Image processing was performed with MATLAB image processing tools (MATLAB R2020b). Cell nuclei were segmented from the DAPI signal color channel by first smoothing the image with a Gaussian filter, then converting the images to binaries by an adaptive threshold and performing a distance field and watershed transform to separate cell nuclei with touching borders. Cell nuclei were separated in a label matrix where pixels belonging to the same nucleus were given the same label/value. Nuclei area and major and minor axis length were sampled for each segmented label.

γ-H2AX foci within the boundaries of the segmented cell nuclei were similarly segmented from the γ-H2AX signal color channel and related to the respective nucleus. To reduce background, a median filtered image was subtracted from the original. Then, the images were smoothed by a Gaussian filter and converted to binaries and foci were individually segmented with a label matrix. Only foci with a minimal area of 9 pixels were segmented.

To exclude cell nuclei in the late stages of the cell cycle, often overexpressing γ-H2AX foci, the following inclusion criteria were used: Cell nuclei with a segmented DAPI surface of 35–209 µm^2^ (2000–12,000 pixels) were considered and nuclei with more than 20 foci were excluded.

As has been shown by Antonelli et al., the size of foci induced by alpha particle tracks can differ both from foci induced by X-rays and from background foci in non-irradiated cells [27]. To evaluate the induction of large foci, a second data set where only foci with an area above 30 pixels were included was generated, in similarity with the method used by Svetlicic et al. [28]. These results are from now on referred to as large foci.

### GATE Monte Carlo simulation

Simulations were performed using GATE [29] (v. 8.0) and Geant4 (v. 10.03). The low-energy electromagnetic physics list constructor *emstandard_opt3* was used in all simulations.

Tools available in the GATE toolkit (Actors) were used to estimate the geometrical efficiency of the PIPS detector and to simulate the energy spectrum and directional distribution of alpha particles reaching the well bottom. Also, the number of alpha particle hits, total energy deposited, and absorbed dose in individual cell nuclei phantom at the bottom of the well was sampled with the GATE Dose Actor. A hit is defined as an alpha particle entering the scoring volume, i.e., the cell nucleus. The energy, LET, and energy imparted per alpha particle reaching a cell nuclei phantom volume were recorded with the GATE Energy Spectrum Actor.

In GATE, the emission from a source can be described by an imported emission spectrum. The normalized detected energy spectrum from the PIPS detector measurement was used to define the emission from the simulated ^241^Am source surface.

The PIPS detector geometrical efficiency was estimated through a simulation of the detector–source geometry to 3.4% (further described in Additional file 1: Fig. S1). Scaling the fluence at the detector surface to the source surface fluence in the PIPS measurements resulted in a surface rate of 2.4*10^5^ alpha particles/second. This fluence was then implemented in the cell irradiation simulations.

### Irradiation setup model

The transport of alpha particles through the well to the bottom and the resulting energy loss in air were simulated by constructing a GATE model of the irradiation setup depicted in Fig. 1, consisting of the well, the source and a 20-µm-high water cylinder, at the well bottom. Visualization of the simulation geometry can be found in Additional file 1: Fig. S2.

The walls of the well were simulated as a 17-mm-high plastic hollow cylinder with an inner diameter of 16.2 mm and a wall thickness of 0.7 mm. The bottom was simulated as a 1-mm-thick plastic cylinder directly under the well walls. The ^241^Am source was simulated as previously described, placed above the well opening with a source-to-bottom distance of 19 mm. The surrounding volume between the well and the source was simulated as air.

Inside the 20-µm water layer, cell nuclei phantoms were placed 2.5 µm below the surface. All cell nuclei were simulated as volumes of water.

Cell irradiation simulations were performed, with total number of primary alpha particles emitted equal to the calculated fluence for 4, 8, and 12 min, as calculated from the source surface dose rate.

### Constructing the elliptical cylinder phantom

**Fig. 2:**
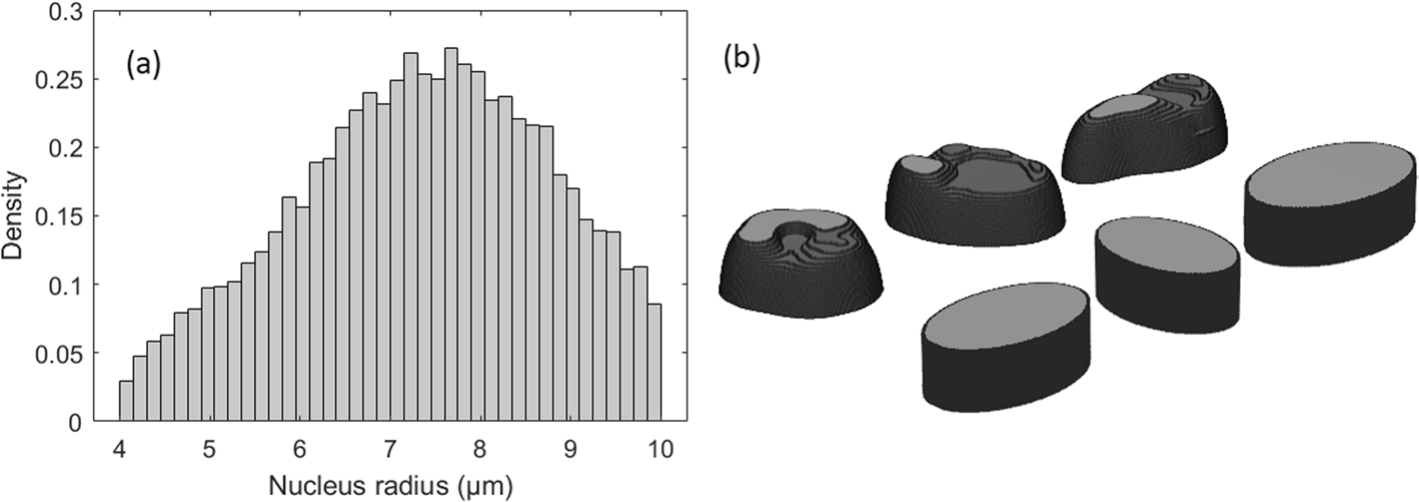
Distribution of major axis lengths of PC3 cell nuclei in elliptical model (**a**). Measured from segmented DAPI-stained cell nuclei. Examples of cell phantom models (**b**). Elliptical cylinder phantoms (front row) generated from the axis lengths in (**a**). Examples of segmented 3D volumes (back row) from confocal imaging of DAPI-stained nuclei

Results from the DAPI image segmentation were used as input in the creation of elliptical cylinder cell nuclei phantoms. A logistic distribution was fitted to the distribution of major axis of the segmented cell nuclei (Additional file 1: Fig. S4). Applying the inclusion criteria for DAPI area stated previously, meant excluding cell nuclei with a major axis length less than 4 µm or longer than 10 µm. The resulting probability distribution of the major axis lengths considered is pictured in Fig. 2a.

Ten thousand elliptical cylinders were created to model as PC3 cell nuclei in the simulations (examples shown in Fig. 2b). The major axis lengths were generated by a random number generator, drawing numbers from the probability distribution. For all elliptical cylinders, the minor axes were 2/3 of its major axis length, and for all the height was 8 µm, which was the mean height of the segmented PC3 cells from the DAPI z-stack (described below). In the simulation, these phantom nuclei were randomly spread out to not overlap each other, in the central 4 × 4 mm^2^ area of the well bottom, to match the center area on the coverslip glass where the fluorescent imaging was performed. This way, the decreasing alpha particle fluence with increasing distance from the well center is considered controlled, as fluorescent microscopy imaging the whole surface of each coverslip would be too time-consuming.

### Constructing mesh volume phantom

3D models of PC3 cell nuclei were segmented from the reconstructed z-stack DAPI signal, examples in Fig. 2. A total of 105 cell nuclei were segmented. The binary voxel matrix was converted to a tessellated mesh surface (stl file format). The mesh volumes were read into the GATE simulation geometry individually as positioned in the original image volume. The image volume was repeated across the well bottom 95 times, thereby generating a total of 9975 cells nuclei phantoms, all within the central 4 × 4 mm^2^ area.

### Statistical analysis

#### Two-sample Kolmogorov–Smirnov test

The control and sham-irradiated cells foci distributions were compared by the nonparametric two-sample Kolmogorov–Smirnov test to investigate if the time cells were without cell culture media-induced γ-H2AX foci. The test evaluates the difference between the cumulative density functions of the two data sets over the total range of both data sets. The null hypothesis assumes that the data sets are from the same continuous distribution. The null hypothesis was rejected at a 5% significance level.

#### Deconvoluting RIF and background foci

The detected γ-H2AX foci in irradiated cells are assumed to consist of radiation-induced foci (RIF) superimposed on an already existing foci background. To estimate the RIF distribution, the probability mass function (PMF) for RIF was deconvolved from the detected foci distribution, assuming that the detected foci in the sham-irradiated cells correctly described this background foci PMF.

For discrete values of $y = 0,1,2,3 \ldots m$, the convolution $p_{z}$ of two PMFs $p_{x}$ and $p_{y}$ is the summation of a series of products of the two underlying PMFs, described as:

1 $$p_{z} \left( z \right) = \mathop \sum \limits_{y = n}^{m} p_{x} \left( {z - y} \right)p_{y} \left( y \right)$$

In this case, $z$ is the number of foci, $p_{z}$ is the probability to detect $z$ number of foci, modeled from the detected foci in irradiated cells, $p_{y}$ is the probability of the background foci, modeled from the resulting detected foci in sham-irradiated cells and $p_{x}$ is the unknown PMF of RIF. From this, $p_{x}$ for *z* = 0–15 was derived, and for each data set of detected γ-H2AX foci the RIF distribution was calculated (derivation further explained in Additional file 1).

#### Calculating simulated foci from simulated hits

Linear regression between mean number of simulated hits and mean detected RIF per cell nuclei was performed. Then, for each simulated hit, the hit was either kept or removed by a probability equal to the slope of the linear fit. This was performed for the large foci, with either elliptical or mesh phantom scoring the hits. After that, the distributions were assumed to represent simulated RIF.

## Results

### Alpha source energy spectrum and fluence

**Fig. 3:**
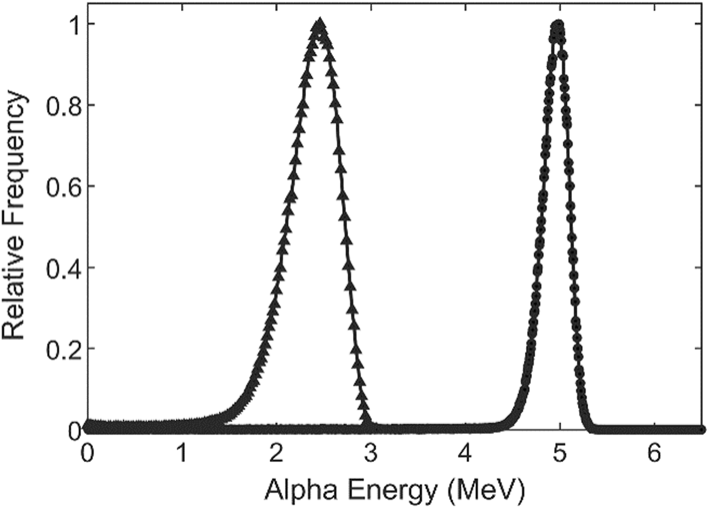
Measured ^241^Am source alpha energy spectra detected by PIPS detector (circles) and simulated energy spectra of alpha particles reaching the bottom of the cell well (triangles)

The detected alpha energy spectra showed a peak energy at 4.9 MeV (Fig. 3). The main alpha emissions from ^241^Am at 5.486 MeV (yield 84.5%) and 5.443 (yield 13.0%) are partly energy-degraded in the source volume before exiting through the source window. These results agree with previously published data [26].

Simulating the transport of alpha particles emitted from the source through the well toward the bottom showed that 7.6% (uncertainty < 0.1%) of particles reached the bottom of the well where the energy loss resulted in a peak energy at 2.4 MeV.

Most simulated alpha particles reaching the well bottom did so close to perpendicular to the well bottom; more than 99% of all particles did so with an angle within ± 18 degrees to the well axis (Additional file 1: Fig. S3a) in Additional file 1). Effectively, this means that the irradiation setup leads to a source collimation and that alpha particles mainly hit cells close to perpendicularly to the cell surface. This limits the probability of foci from separate alpha particle tracks overlapping each other along the well axis. Also, simulation showed that alpha particle energy was deposited across the bottom surface with a slight gradient from the center of the well bottom toward the walls of the well (Additional file 1: Fig. S3b). This, as previously described, will put adherent cells at varying probability to be hit by an alpha particle as a function of its distance from the center of the well. To compensate for this, cell nuclei were only imaged in the central 4 * 4 mm^2^ square of the cover slip.

### Foci distribution

A total of 12,823 control nuclei, 4805 sham-irradiated nuclei, 6789 nuclei irradiated 4 min, 5073 nuclei irradiated 8 min, and 7355 nuclei irradiated 12 min were segmented from the DAPI signal. For the control cell nuclei, the mean diameter of the major axis length was 15.3 ± 4.1 µm (Additional file 1: Fig. S4). For all nuclei, foci were segmented from the γ-H2AX signal.

**Fig. 4:**
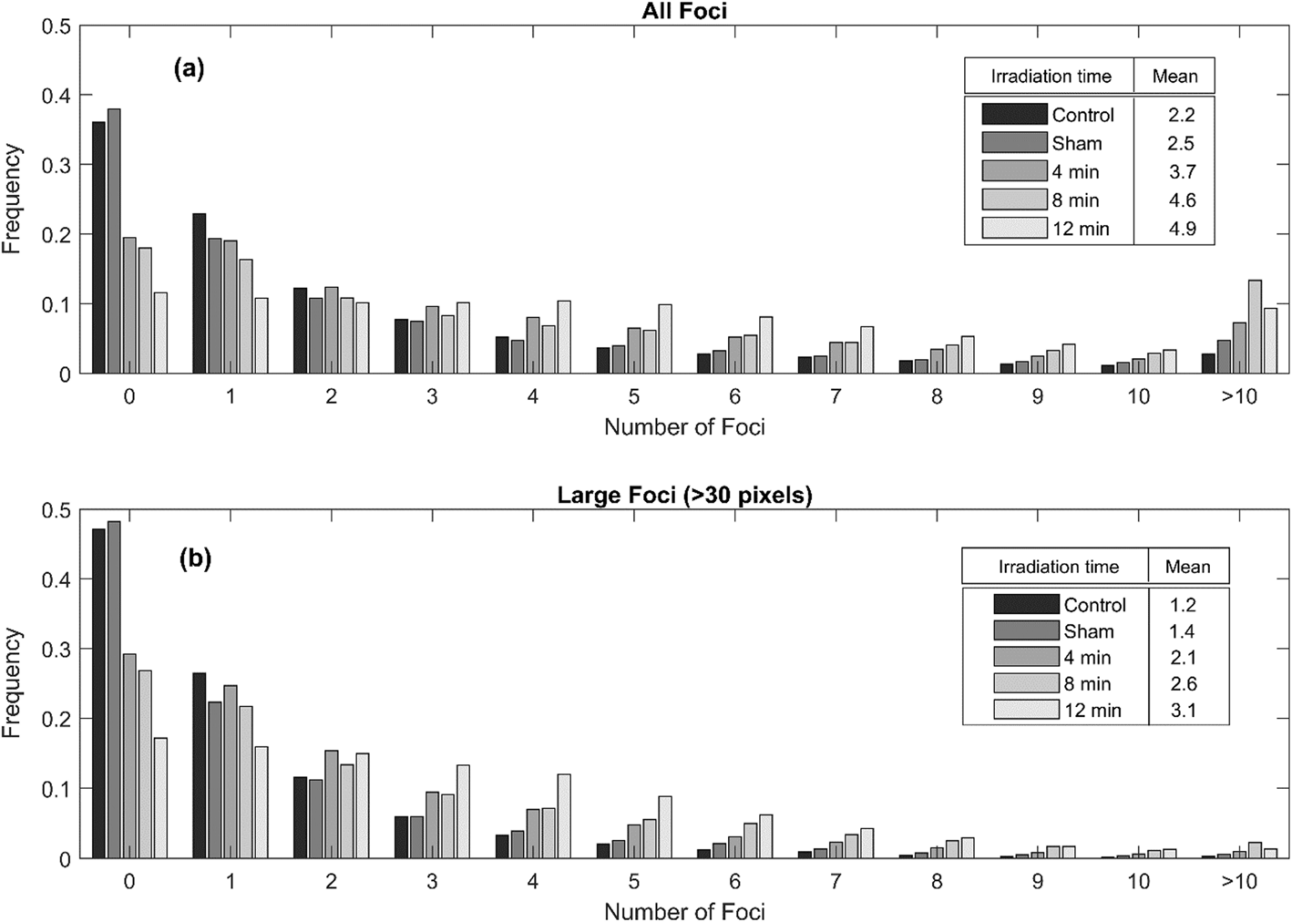
Detected γ-H2AX foci distribution per nucleus after alpha irradiation for 4, 8, and 12 min with the ^214^Am source. In sham-irradiated cell, the cell culture media was removed for 12 min. **a** Detected distribution considering foci larger than 9 pixels. **b** Detected distribution considering foci larger than 30 pixels

The detected γ-H2AX foci, summarized in Fig. 4a, showed an increased mean number of foci per cell for increased irradiation time. PC3 cells, like many other cancer cell lines, have quite a high background of γ-H2AX foci. An average of 2.2 foci per cell nuclei was, however, a lower background compared to 4.7 as previously reported for PC3 cells by Sedelnikova and Bonner [30]. Excluding foci with an area less than 30 pixels, summarized in Fig. 4b, lowered the background in controls to 1.2 foci per nucleus.

The two-sample Kolmogorov–Smirnov test comparing control and sham-irradiated nuclei found a significant difference between the control and sham-irradiated distributions (*p* < 0.01). The difference remained significant also when only considering large foci (*p* < 0.01).

The mean area of the segmented foci increased with irradiation time (Additional file 1: Fig. S5). This can both be a result of alpha particle induced foci being larger than background foci, and an effect of foci clustering. For longer irradiation times, there is an increased probability for foci clustering, thus multiple foci being segmented as a single larger foci. It might also be reflected in the fact that the variance of detected foci increased between 4 and 8 min of irradiation time, while detected foci after 12 min irradiation had a lower variance than both 4 and 8 min (data not shown) when considering all foci. When only considering large foci, the variance was continuously increasing with irradiation time.

### Simulation results

**Fig. 5:**
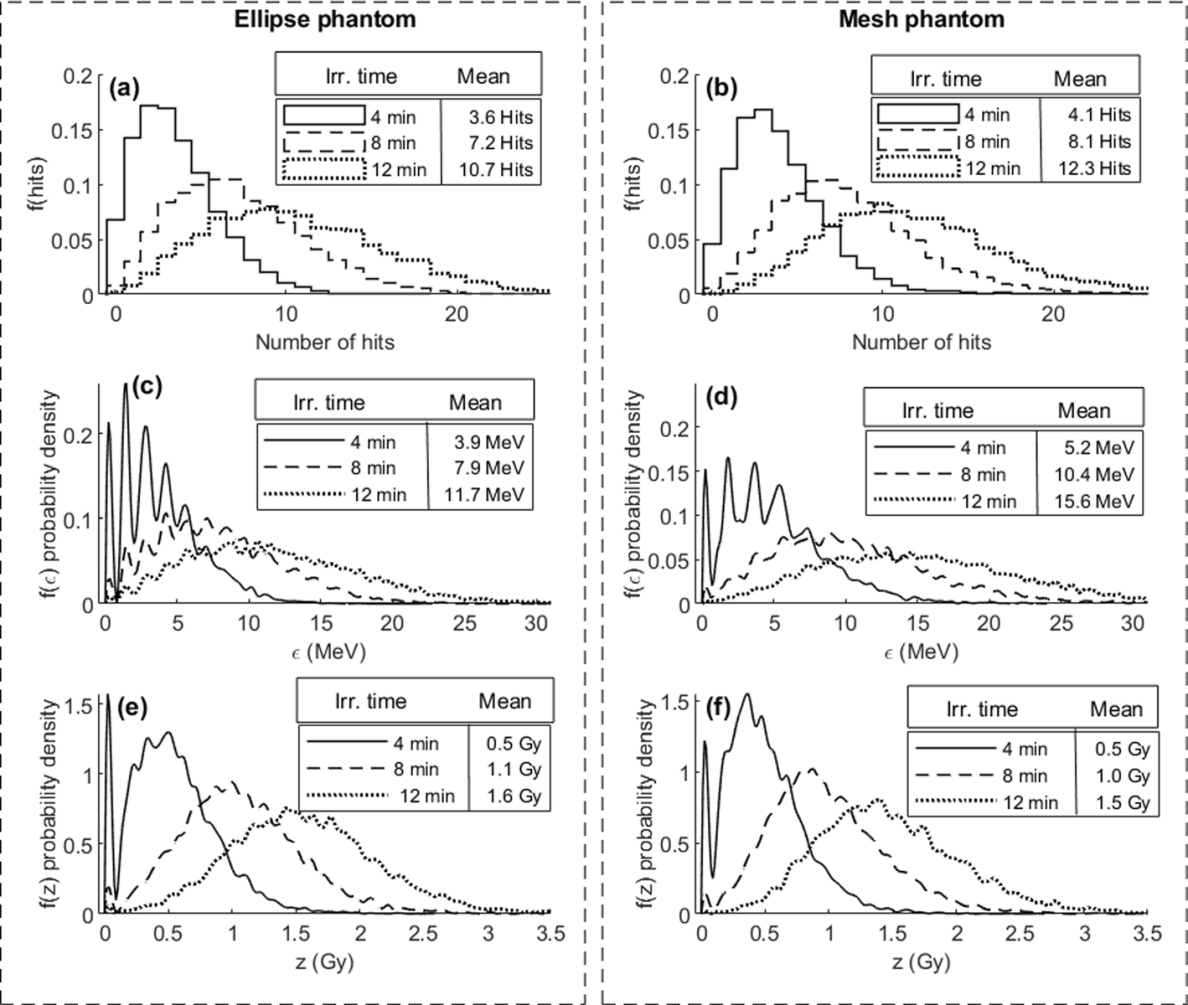
Simulation results for elliptical cylinder phantom (left panel **a**, **c**, **e**) and the mesh volume phantom (right panel **b**, **d**, **f**) figured as probability density functions (PDFs). The number of alpha particle hits per cell nuclei, f(hits), in **a** and **b**, the energy imparted per cell nuclei PDF, f(ε) in **c** and **d**, and the specific energy per cell nuclei PDF, f(z), in **e** and **f** for respective phantom

The alpha source irradiation simulations of model PC3 nuclei phantoms are summarized in Fig. 5. Here, zero to multiple alpha particles have hit individual cell nuclei and the sum of their interactions forms the results. The number of alpha particle hits, $f\left( {{\text{hits}}} \right)$ (Fig. 5a, b), energy imparted, $f\left( \varepsilon \right)$ (Fig. 5c, d), and specific energy, $f\left( z \right)$ (Fig. 5e, f), probability density functions (PDF), for the elliptical cylinder phantom and the mesh phantom for the equivalent of 4, 8, and 12 min of irradiation were simulated. The elliptical cylinders resulted in a slightly lower mean number of alpha particle hits and energy imparted per nuclei than the mesh phantom. This can be explained by the slight difference in size of the two phantoms, the elliptical cylinders mean volume being 930 µm^3^, while the mesh phantom nuclei mean volume was 1070 µm^3^. The resulting variance and mean of the specific energy from the two phantoms were, however, very similar. In both phantoms, the energy imparted probability densities show the characteristic peaks resulting from nuclei experiencing no or few alpha tracks. In Fig. 5c, d, peaks representing 0–4 alpha particle hits per nuclei can be clearly distinguished. When the mean number of hits per cell nuclei is low, the difference between the energy imparted from one or a few alpha particles is more or less multiples of the average energy deposited by a single hit. As the irradiation time is increased, the mean number of hits increases and most of all cell nuclei are hit with multiple alpha particles. The average energy imparted distribution is then “smoothed,” and the peaks become less pronounced.

**Fig. 6:**
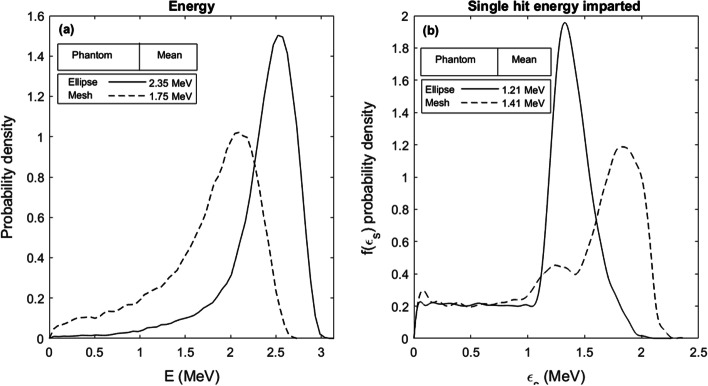
Simulated energy (**a**), and energy imparted (**b**) PDFs per alpha particle hitting any of the cell nuclei volumes in the elliptical cylinder phantom or the mesh volume phantom, respectively

This simulation setup and the tools available in GATE allow estimation of the energy of the alpha particles as they enter the nuclei volumes, as summarized in Fig. 6a. Results are shown for both the elliptical cylinder phantom and the mesh volume phantom. The difference in mean alpha energy seen between the phantoms is mainly due to small variations in source-to-target distances. The elliptical cylinder nuclei are all the same height (8 µm), while the mesh volume nuclei vary slightly along the cell well axis. Therefore, these high LET alpha particles at the end of their tracks quickly loose energy and small variations in distance gives a large impact. Also, the energy imparted per alpha particle track, also known as the single-hit energy imparted, $\varepsilon_{{\text{s}}}$, is shown in Fig. 6b. Differences between the phantoms shape, volume, and height again impact the possible alpha particle track lengths through the target volumes. The elliptical cylinder phantoms display a continuous plateau before a single distinct peak, while the mesh volume phantoms have two distinct peaks. This is likely the reflection of a more continuous distribution of size and shape in the elliptical cylinder phantoms, while the small sample of 105 segmented cells forming the basis of the mesh volume phantom might be more unevenly distributed.

### RIF distribution

The detected RIF probability density was calculated through deconvolution of the detected large foci distributions PDF in irradiated cells and sham-irradiated cells. Sham-irradiated cells, as previously shown, differed significantly in number of foci from the control, why the distribution from sham-irradiated cells needs to be considered in the deconvolution to also consider foci induced due to the removal of the cell medium during the irradiation. As summarized in Table 1, the mean number of large RIF for 4, 8, and 12 min of irradiation was 0.7, 1.2, and 2.25 foci per nucleus, respectively.

**Table 1:** Summarized properties of the detected RIF distribution, and the simulated RIF distributions from the elliptical cylinder phantom and the mesh volume phantom

Irradiation time	Data	Mean	Median	Mode	Variance	SD	CV
4 min	Detected RIF	0.7	0	0	1.2	1.1	0.6
	Simulated ellipse RIF	0.7	0	0	0.8	0.9	0.8
	Simulated mesh RIF	0.7	0	0	0.9	0.9	0.8
8 min	Detected RIF	1.2	0	0	3.6	1.9	0.6
	Simulated ellipse RIF	1.4	1	1	1.8	1.3	1.1
	Simulated mesh RIF	1.4	1	1	1.9	1.4	1.1
12 min	Detected RIF	2.2	2	0	3.7	1.9	1.2
	Simulated ellipse RIF	2.1	2	1	2.8	1.7	1.3
	Simulated mesh RIF	2.2	2	1	3.1	1.8	1.3

**Fig. 7:**
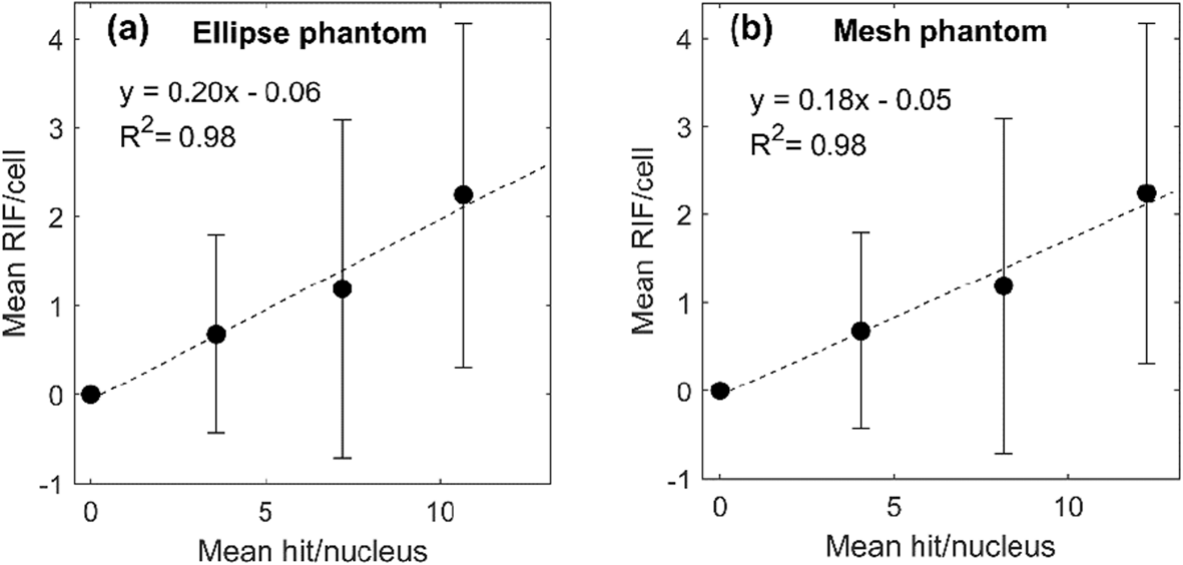
Mean number of RIF per cell nuclei as a function of simulated mean number of hits per cell nuclei for the elliptical cylinder phantom (**a**) and the mesh volume phantom (**b**), respectively. Error bar 1 +—SD. Linear regression by linear least squares method seen as dotted line

The linear fit between detected RIFs and simulated hits for the elliptical cylinder phantom (Fig. 7a) was used to estimate a scaling factor between simulated hits and simulated foci. On average, 5 simulated hits were needed to induce one RIF. The slope of the linear fit when considering simulated hits in the mesh phantom was very similar to the elliptical phantom (Fig. 7b) (0.18 (0.11–0.30 95% CI) vs 0.20 (0.10–0.26 95% CI)). The large SD, as shown by the error bars, is an expected effect of the large variance of the mean number of alpha particle hits experienced by the irradiated cells, as shown by the simulation results in Fig. 5a, b. The size of this variance will depend on the irradiation setup geometry and is therefore necessary to model to perform accurate small-scale dosimetry. It gives an opportunity to estimate the proportion of cell nuclei reaching a certain number of hits or absorbed dose, which might be a better dosimetric unit than the mean absorbed dose when comparing to a biological response.

**Fig. 8:**
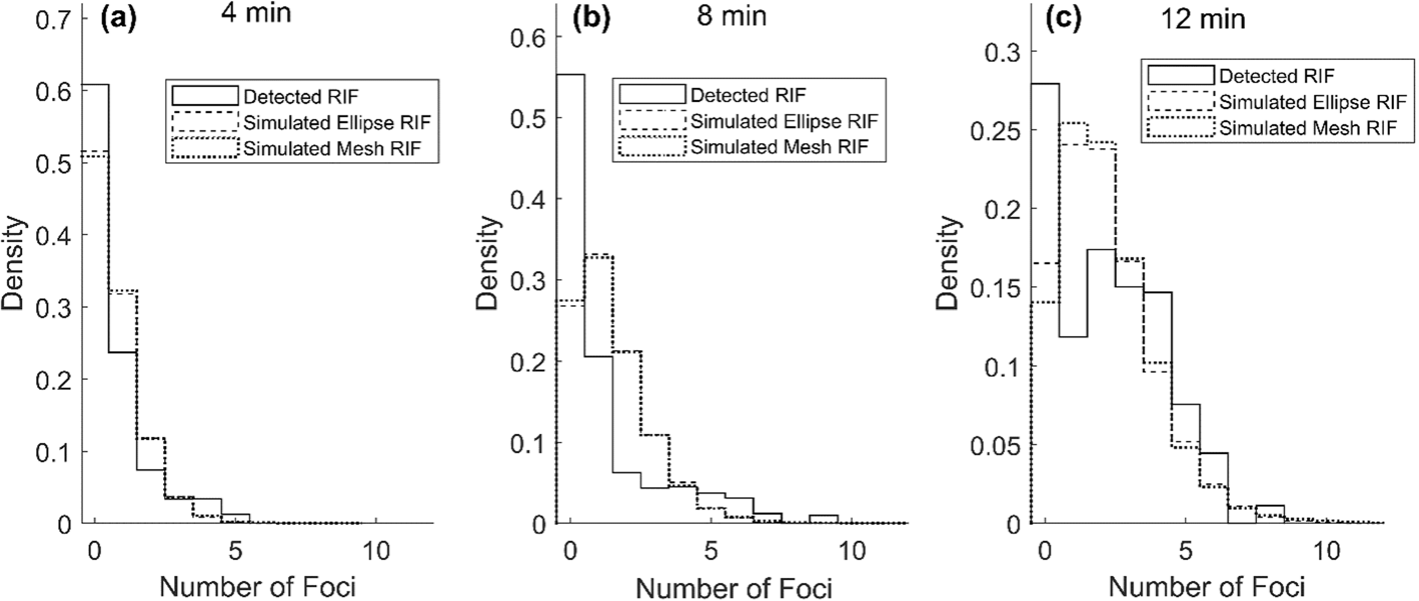
Detected RIF distribution compared with simulated RIF distributions for the elliptical cylinder phantom and mesh volume phantom, respectively. Results for 4 min (**a**), 8 min (**b**), and 12 min (**c**) of alpha ^241^Am source irradiation

The simulated hits distributions were rescaled as previously described. The resulting simulated RIF distribution from the elliptical cylinder phantom and the mesh volume phantom is compared to detect RIF after 4, 8, and 12 min of alpha irradiation in Fig. 8 and in Table 1.

As summarized in Table 1, means of the distributions are in good agreement, while the standard deviation (SD) of detected RIF is consistently higher than simulated foci. The detected RIF distributions have a somewhat zero-inflated shape, with a higher probability for zero RIF than would be expected by the simulations. This is especially pronounced for detected RIF after 8-min irradiation and are assumed to be due to limitations in the imaging-to-segmentation workflow.

## Discussion

The short range of alpha particles and their high LET make it necessary to consider microscopic targets when performing dosimetry. Otherwise, the damage distribution and the expected biological outcome might be greatly misestimated. The main cause of cell death from ionizing radiation is double-strand breaks on the DNA. In both tissue and in vitro cell cultures, multiple cells or cell nuclei might not experience any traversing alpha particles in cases where a macroscopic dosimetry model would report a significant absorbed dose. Therefore, if only macroscopic volumes are considered in the model, large variances of hitting particle tracks, energy imparted, and specific energy in microscopic volumes, such as the cell or cell nucleus, will not be detected. To test this, we hypothesized that the distribution of radiation-induced γ-H2AX foci should follow the same distribution as the simulated alpha particle hit distribution in phantom cell nuclei in a model of the irradiation setup.

This study is partly limited by the fact that only a single plane through the center of the cell nucleus is imaged to detect γH2AX foci. There is a risk that foci under or above this level will be missed. Others have shown that foci in a thin cell nucleus extend most of the nucleus height, meaning single central planes are likely to detect all existing foci [31]. Also, alpha particles induce larger foci along the particle track [27] which would be expected to appear in multiple planes.

**Fig. 9:**
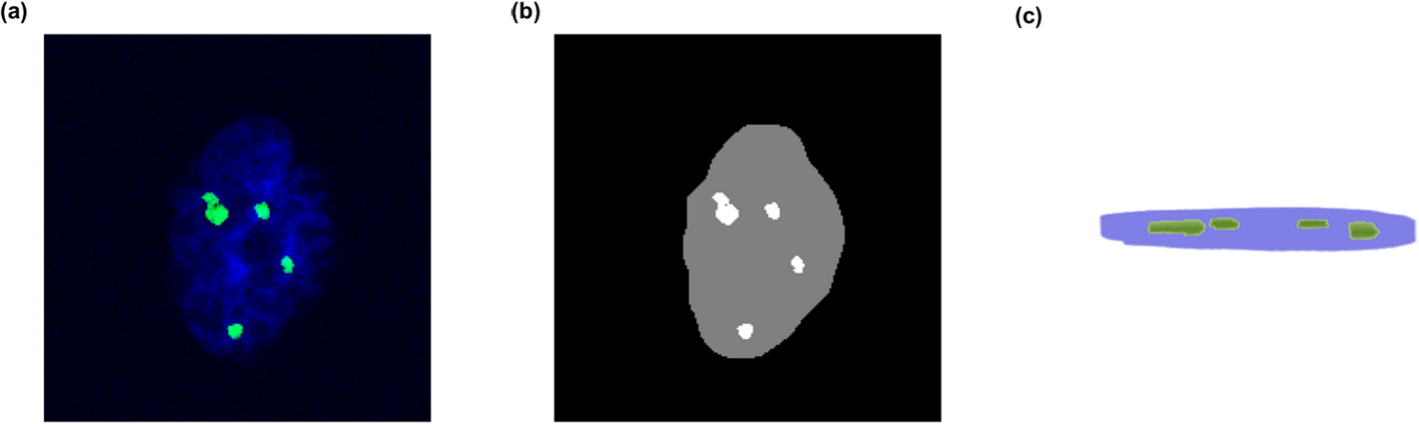
Assessing the risk of missing foci when only a single plane centered in the cell nuclei is imaged. In (**a**), representative γH2AX foci in a single plane from the z-stack are detected. Segmented foci (**b**) in a single plane and (**c**) a 3D representation of the detected foci in the full volume of the nucleus

The zero-inflated shape of the detected foci distribution (Fig. 8 and Table 1) could, however, be explained by undetected foci. To evaluate this, we investigated the small cohort of confocally imaged cells that were initially imaged to generate the mesh phantoms. The risk of missing foci when only a single plane centered in the cell nuclei is imaged was therefore assessed in 89 confocally imaged cell nuclei. In Fig. 9a, representative γH2AX foci in a single plane from the z-stack are detected. The foci were segmented (Fig. 9b) in all planes of the z-stack, and the number of detected foci in the most central plane of the nucleus was compared to the number of foci detected in the full volume of the nucleus (Fig. 9c).

Although most foci were detected in a central plane, they constituted approximately 75% of the foci detected in the full volume (Additional file 1: Fig. S7).

**Fig. 10:**
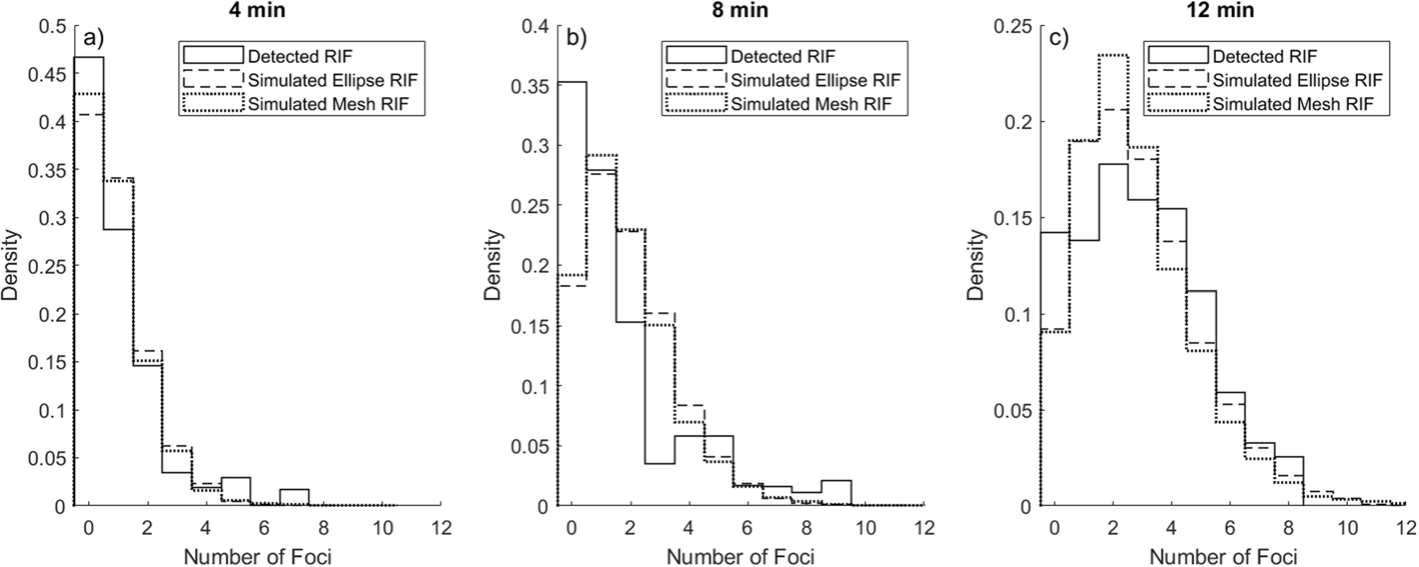
Detected RIF distribution compared with simulated RIF distributions after adjusting for missed foci for the elliptical cylinder phantom and mesh volume phantom, respectively. Results for 4 min (**a**), 8 min (**b**), and 12 min (**c**) of alpha ^241^Am source irradiation

These undetected foci seem to be randomly distributed among cells with, no, a single or several foci detected in the single plane; however, this is uncertain due to the low number of cells investigated. Assuming the random distribution true, the expected portion of undetected foci in each cohort were added by a random number generator, with all segmented cell nuclei having an equal probability of receiving extra foci. This improved the similarity between the detected and simulated foci as shown in Fig. 10 and Additional file 1: Table S1.

We believe this indicates that foci truly were missed in our study and that this correction offers a method to extrapolate from a single plane to the full nuclei volume and allows an accurate estimate of the foci distribution in the full nuclei volume, even if a single plane is imaged.

We have used two different methods to generate cell nuclei phantoms to simulate the number of hits, energy imparted, and specific energy in PC3 cell nuclei. For both models, fluorescence microscopy imaging of DAPI-stained cell nuclei served as input to the models. In the first case, an elliptical cylinder was assumed as a good estimate of the shape of an adherent PC3 cell nucleus. Measuring the axis lengths of segmented cell nuclei is easily automated in an image processing workflow, and many segmented cell nuclei could easily be included in the estimate of the major and minor axis length distributions. The model was further simplified assuming a fixed relationship between the minor and major axis, with the minor axis being two-thirds of the major axis in every model nucleus. In the second model, DAPI-stained cell nuclei were imaged as a z-stack, which generates a 3D image of the nuclei. As multi-focal plane imaging is very time-consuming, only a smaller set of cells were imaged, resulting in a final number of 105 PC3 cell nuclei being segmented. The segmented 3D volumes were repeated on the well bottom in the simulation geometry to cover the relevant surface. The smaller sample size risks not correctly represent the size variation. The segmented 3D volumes were more irregular in shape than the simpler elliptical cylinders and on average had a larger volume, generating more hits and a higher mean energy imparted for the same simulated irradiation times (Fig. 5a–d). However, the simulated specific energy distributions were in good agreement. For targets of the size of cell nuclei, alpha particle track generates energy depositions proportional to the track length through the nuclei volumes. The on-average larger mesh volumes most likely experienced longer track lengths, but the resulting energy imparted was then divided by an on-average larger mass than those in the elliptical phantom. This might explain the similar results for the specific energy.

In both models, the nucleus size and shape are estimated from the DAPI stain, and they are therefore similarly limited by the risk that this does not correctly represent the true size of the cell nucleus. This is a similar method to that used by Bareret et al. [25]. We measured the mean diameter of the major and minor PC3 cell nucleus to be 15.3 µm and 10.1 µm, respectively (Additional file 1: Fig. S4). This is slightly larger than the results of Moore et al. who found the mean PC3 nucleus diameter to be 12.2 µm, measured with an imaging flow cytometer [32]. However, considering that cells and nuclei take a more spherical shape while in suspension, our results agree with these measurements.

Also, in difference to other studies [31, 33], we did not attempt to exclude cells outside of the G0/G1 cell cycle phase. Instead, we excluded the largest and smallest segmented nuclei, to limit errors of the staining and imaging process, the automated segmentation, and excluded cells with and excess number of foci (> 30), likely to be in active mitosis. Since size and morphology of cells grown in vitro seem to be very dependent on seeding and growth conditions, it seems necessary to independently assay the size and shape to generate a representative model for the specific irradiation conditions of an experiment.

The ^241^Am source used here has previously been described by and modeled for a cell irradiation setup in GATE by Nilsson et al. [26]. Their GATE simulation model did not include a cell or cell nuclei model but considered the absorbed dose in a 20-µm-high water cylinder at the bottom of a cell well insert. They estimated the source activity to about 400 kBq with HPGe gamma spectroscopy [26]. This, however, does not directly give the energy spectrum and emission rate at the surface of the source, as the magnitude of self-absorption is unknown. Therefore, they modeled the internal activity distribution inside the source volume and simulated the transport of alpha particles to the source window. Matching this to a measured alpha particle spectrum functioned as a control for when the model was correct. We instead used an energy spectrum and the fluence to describe the emissions from a source plane placed at the source volume window in the simulation geometry. This eliminates the need to know the exact activity and its distribution within the source volume which simplifies the simulation.

Cell and cell nuclei models have been implemented in Monte Carlo simulations for dosimetry simulations by multiple groups [19–21, 23–25, 33, 34]. The results in Figs. 5 and 6 represent the cell nuclei population, taking the variance in cell nuclei size into account in the probability densities. More often, and by the conventions of microdosimetry [1, 10, 13], the energy deposited and specific energy probability densities are calculated for a single given volume, often a cell, nucleus, or other organelle. As our aim was to correlate the resulting hits with the detected γ-H2AX distribution, size variations would influence the possible number of both hits and resulting detectable foci per nucleus. The 3D volumes of the mesh phantom have an opportunity to better represent the shape of the cell nuclei than a simplified phantom like the cylindrical ellipses. However, we found these easier to construct and implement in a simulation.

We estimated the RIF distribution by assuming that the γ-H2AX foci distribution found in sham-irradiated cells correctly described the background on which the RIF are then superimposed. By deconvolution of their probability densities, the detected RIF probability density was estimated for 4, 8, and 12 min of irradiation. These were compared to the simulated RIF probability densities. Since the simulated RIF were scaled by the slope of the linear fit between the mean detected RIF and mean number of simulated hits in the cell nuclei, it is no surprise that the means of simulated and detected RIF are in good agreement. However, the shape, as described by the moments of the probability densities, should be considered to evaluate if the simulation is capable to correctly estimate the expected damage distribution. Of particular interest is the expected number of cell nuclei expressing zero RIF and the dispersion of the distribution. As shown in Fig. 8, the probability for zero RIF is consistently lower in the simulated nuclei than in the detected. The most probable explanation for the zero-inflated shape of detected RIF lies in the limitations of the operation of the fluorescence microscope and the later post-processing of the images to segment cell nuclei and distinct foci.

Likewise, the standard deviation of the simulated probability density functions is slightly lower than for the detected RIF probability density (Table 1). If the probability to be hit varies greatly among target volumes (cell nuclei), the dispersion of the hit distribution will be large, as will the simulated RIF. The ability of the simulation to correctly model this should be reflected in its conformity to the detected RIF probability density.

We detected large foci by excluding foci with an area less than 30 pixels. Only considering large foci lowered the expected number of foci per alpha track hit, as the number of background foci was reduced in control and sham-irradiated cells. As has been previously shown by others, there is a difference in size between foci induced by X-rays compared to alpha particles. Foci from alpha particles are likely to be clusters of several DNA DSB or a more complex damage site, while small foci from X-rays are more likely to be individual DSB for doses up to 1 Gy [27]. This is then reflected in the difference in repair dynamics, where foci from low LET are repaired and removed more quickly, while high LET foci last over days [27, 35]. To illustrate the size difference, PC3 cells were irradiated by X-rays and stained and imaged as previously described for DAPI and γ-H2AX. A comparison between control, X-ray-irradiated, and alpha particle-irradiated PC3 cells is seen in Additional file 1: Fig. S6. X-ray-induced foci are clearly smaller, while alpha particles generate large spots, more likely to touch each other’s borders and blend to bigger patches. The segmentation of individual γ-H2AX foci might therefore be more complex than for X-rays, more so for uncollimated source irradiations than for precision microbeams, where multiple alpha particles can be aimed at different positions in the cell nuclei to avoid foci overlap [33]. As reported by Horn et al., alpha particles have been seen to induce a pan-nuclear phosphorylation of H2AX [36], leading to an increased fluorescent background signal in the cell nuclei outside of foci. This was not seen in X-ray-irradiated cells and is supposed to be an effect from high LET irradiation.

Other groups have investigated the probability for overlapping γ-H2AX foci along the alpha particle tracks hitting the nucleus perpendicular to the surface [27, 33]. These could then be mistaken for a single focus if only a single focal plane is imaged. Antonelli et al. found it hard to distinguish individual foci in human fibroblast cell nuclei with an average height of 3 µm when irradiated with 125.2 keV/µm alpha particles, even though simulations estimated a mean of 8 DSB per track; hence, they assumed each focus observed perpendicular to the surface corresponded to an average of 8 DSB. This is a limitation in the use of γ-H2AX as a marker for DSB induced by high LET radiation. However, for the type of irradiation geometry used in this study, it potentially leads to a linear relationship between number of tracks passing through the nucleus and detected foci. This can also be used as a tool for radiation dosimetry, as it could be used to estimate the proportion of irradiated cells nuclei not traversed by a single alpha particle.

Gonon et al. investigated the induction of γ-H2AX foci in HUVEC cells irradiated with alpha particles through the cell nucleus by a microbeam [33]. Clusters of simulated DSB were compared to detect γ-H2AX foci in cells hit with five alpha particles per nuclei. They showed an increased frequency of induced focus per track with increasing LET (decreasing particle energy) with an apparent plateau for high LET. For 1.86 MeV alpha particles, the probability to form at least one focus was estimated to be 0.69. For 5.5 MeV alpha particles, the estimated frequency was 0.59. In this paper, we calculated the slope between detected RIF and simulated hits in the phantom nuclei to 0.20 for the cylindrical ellipse phantom and 0.18 for the mesh volume phantom, as an estimate of the probability for a hit to induce a detectable RIF. This lower frequency could be explained by (except for differences in radiosensitivity between cell lines) the larger variance in energy deposited in cells irradiated by an uncollimated source surface compared to cells irradiated with a microbeam aiming at the nucleus. In Fig. 6c, the simulated single-hit energy imparted shows a peak at 1.4 MeV for the elliptical cylinder phantom and 1.8 MeV for the mesh phantom, but a distinguishable tail toward lower energies for both phantoms, representing alpha particles not taking the longest possible path through the nucleus. For the elliptical cylinder phantom, approximately ¼ of all alpha particles deposit an energy less than 1 MeV, making up the plateau below the peak. For a collimated microbeam, the energy imparted PDF would consist of a narrow single peak, and hence all alpha particles delivered would have a similar probability to generate a detectable γ-H2AX focus. For irradiations with uncollimated sources, the probability will vary more.

## Conclusion

In this study, we have developed a small-scale dosimetry model that predicts the damage distribution measured as the γ-H2AX foci distribution in cells irradiated with an alpha-emitting source. The Monte Carlo dosimetry simulations revealed a large variation of number of alpha particle hits, energy imparted, and specific energy experienced by the cell nuclei volumes. Our results emphasize the need to carefully consider microscopic targets for short-range, high LET radiation, such as alpha particles, to reliably estimate dosimetric quantities.

## Supplementary Information

**Additional file 1.** Supplemental material.

## Data Availability

The data sets used and analyzed during the current study are available from the corresponding author on reasonable request.

## Abbreviations

RNT: Radionuclide therapy
TAT: Targeted alpha therapies
DSB: Double-strand breaks
RIF: Radiation-induced foci
LET: Linear energy transfer
PDF: Probability density functions
PMF: Probability mass function
SD: Standard deviation
CI: Confidence interval
