# Supplementary material for: Small-scale dosimetry for alpha particle 241Am source cell irradiation and estimation of γ-H2AX foci distribution in prostate cancer cell line PC3
Source: EJNMMI Phys. 2022 Jul 19;9:46. doi: 10.1186/s40658-022-00475-x (PMC9296737; doi:10.1186/s40658-022-00475-x)
Supplement: Supplementary file 1 — Additional file 1. Supplemental material. [file 40658_2022_475_MOESM1_ESM.docx]

**Supplemental data – Small-scale dosimetry for alpha particle 241Am source cell irradiation and estimation of γ-H2AX foci distribution in prostate cancer cell line PC3**

**GATE Monte Carlo simulation**

GATE has s collection of tools called Actors, a form of detectors that can be connected to volumes in a simulation to score interactions from the simulated radiation. These were used for several purposes in the simulations performed.

- An Energy Spectrum Actor was used in the simulation estimating the geometrical efficiency of the PIPS detector.
- A Phase Space Actor was used to sample the energy and direction of alpha particles transported from the source surface to the well bottom. (The results from this simulation were used as a new source description in the cell phantom simulations.)
- Dose Actors were used to sample the alpha particle number of hits, energy deposited and absorbed dose in all cell nuclei phantoms.
- An Energy spectrum Actor was used to measure energy, LET and energy imparted by individual alpha particles entering the cell nuclei phantoms.

Filters can be applied to Actors to select a particle type, energy etc. that are to be recorded by the Actor.

Cell phantoms and their attached Dose Actors were handled in separate scripts and called by the main simulation script.

**PIPS detector simulation**

To construct a model of the ^241^Am source, the energy spectrum of the alpha particles detected by the PIPS detector was normalized and used to define the energies of alpha particles emitted from a circular plane with diameter 11.8 mm, such as the source active area. Alpha particles were emitted in a 2*π* solid angle from the front side of the plane facing the detector. The aluminum edge around the source window at the front of the source was simulated as a 1 mm high aluminum cylinder with a 1 mm wall thickness.

The detector was not directly modeled, since all alpha particles reaching the detector can be assumed to be detected. Therefore, a simulation of the plane source and an Energy Spectrum Actor, with an alpha particle filter, placed at 36 mm from the source plane was performed to score the particles reaching the detector and validate that the emitted and detected alpha spectrum were overlapping.


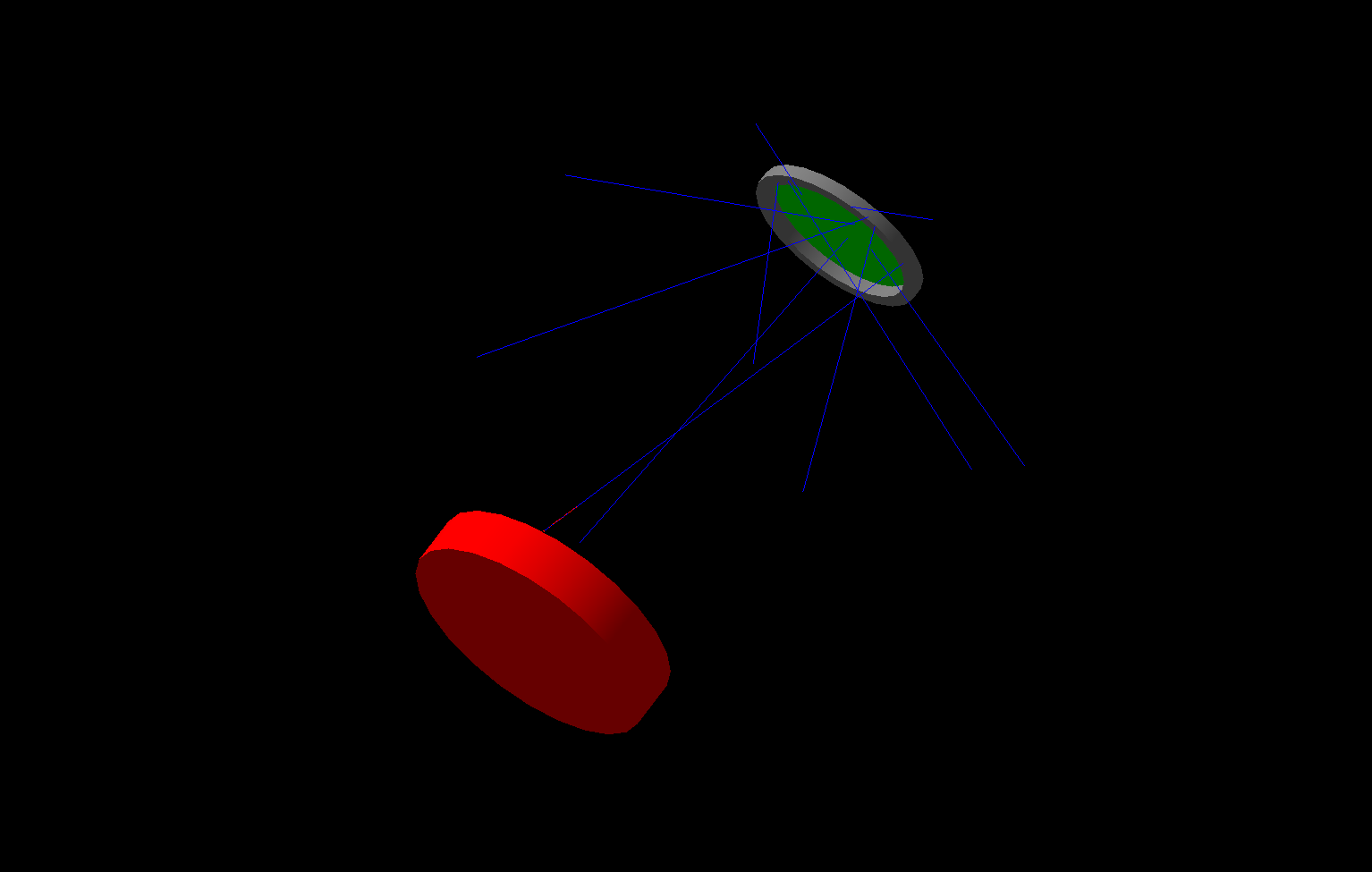


*Figure S1. In the GATE detector simulation alpha particles are emitted isotropic in a 2π semi-sphere from the source surface (green). The detector is simulated as a cylinder of Si02 (red). All alpha particles reaching the detector volume are assumed to be detected.*

**Irradiation set-up model**

A Phase Space Actor defined as a plane was placed 20 µm above the well bottom (just above the water volume) to record the energy and direction of alpha particles reaching the bottom of the well. Particles hitting the walls of the well were killed in the simulation.


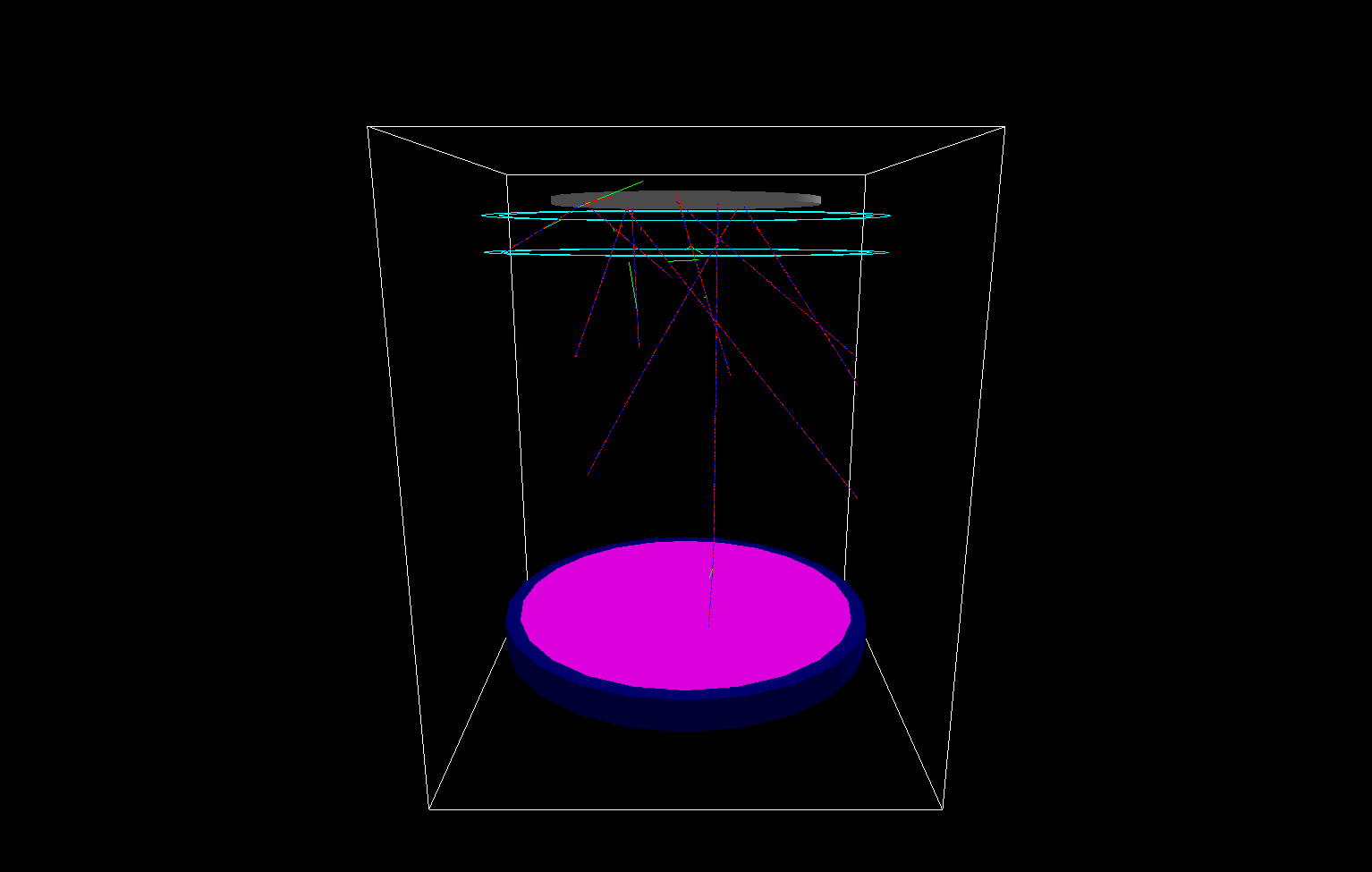


Figure S2. *Visualization of the simulation geometry*. *Alpha source (gray cylinder) is placed above the well opening. The surface of the cylinder facing the well is emitting alpha particles towards the well bottom. Well walls (not visible) are simulated as a plastic hollow cylinder. Well bottom (blue) is simulated as a plastic cylinder at a 19 mm distance to the source. A Phase Space Actor (pink) plane measuring the alpha particles reaching 19 mm into the well is placed above the well bottom. Alpha particles are visualized as blue lines and secondary electrons as red.*


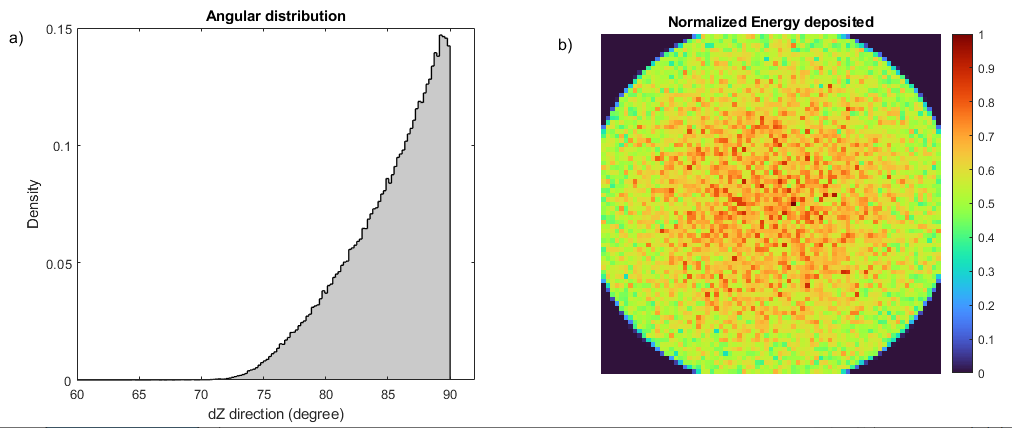


*Figure S3 a) Angular distribution of simulated alpha particles reaching bottom of well and b) the normalized energy deposited at the bottom of the well showing a nun-uniform distribution with a decreasing gradient towards the edge. This is due to the distance between source and target, and that the source surface is smaller than the target surface.*

**Deconvoluting RIF and background foci**

The detected foci distribution in irradiated cells is assumed to consist of the distribution of radiation induced foci (RIF) superimposed on an already existing distribution of background foci. To estimate the RIF distribution, the probability mass function (PMF) for RIF was deconvolved from the detected foci distribution, assuming the detected foci in the sham irradiated cells correctly described this background foci PMF.

For discrete random values, the convolution *pz* of two PMFs *px* and *py* is the summation of a series of products of the two underlying PMFs, described as:

$$p_{z}\left( z \right)=\sum_{y=n}^{m} p_{x}\left( z-y \right)p_{y}(y)$$

$$y=0,1,2,3\ldots m$$

$$p_{y}\left( y \right)=\left\{ \begin{aligned} 0-1, &y\geq0 \\ 0, &y<0 \end{aligned} \right.$$

$$p_{x}\left( x \right)=\left\{ \begin{aligned} 0-1, &x\geq0 \\ 0, &x<0 \end{aligned} \right.$$

In this case, $z$ is the number of foci, $p_{z}$_,_ is the PMF to detect $z$ number of foci, modelled from the detected foci in irradiated cells, $p_{y}$ is the PMF of the background foci, modelled from the resulting detected foci in sham irradiated cells and $p_{x}$ is the unknown pmf of RIF.

$$p_{z}\left( 0 \right)=p_{x}\left( 0-0 \right)p_{y}\left( 0 \right)+p_{x}\left( 0-1 \right)p_{y}\left( 1 \right)\ldots p_{x}\left( 0-y_{m} \right)p_{y}\left( y_{m} \right) =p_{x}\left( 0 \right)p_{y}\left( 0 \right)+0*p_{y}\left( 1 \right)\ldots0*p_{y}\left( y_{m} \right)=p_{x}\left( 0 \right)p_{y}\left( 0 \right)$$

$$\to$$

$$p_{x}\left( 0 \right)=\frac{p_{z}(0)}{p_{y}(0)}$$

$$p_{z}\left( 1 \right)=p_{x}\left( 1-0 \right)p_{y}\left( 0 \right)+p_{x}\left( 1-1 \right)p_{y}\left( 1 \right)+p_{x}\left( 1-2 \right)p_{y}\left( 2 \right)\ldots p_{x}\left( 1-y_{m} \right)p_{y}\left( y_{m} \right)=p_{x}\left( 1 \right)p_{y}\left( 0 \right)+p_{x}\left( 0 \right)p_{y}\left( 1 \right)+0*p_{y}\left( 2 \right)\ldots0*p_{y}\left( y_{m} \right)=p_{x}\left( 1 \right)p_{y}\left( 0 \right)+p_{x}\left( 0 \right)p_{y}\left( 1 \right)$$

$$\to$$

$$p_{x}\left( 1 \right)=\frac{p_{z}\left( 1 \right)-p_{x}\left( 0 \right)p_{y}(1)}{p_{y}(0)}$$

And so on until $p_{x}(y_{m})$ have been calculated (cutoff at $p_{x}(p_{x}<0.01) = 0$).

**Segmented PC3 cell nucleus size**

DAPI stained PC3 cell nuclei were segmented from the fluorescent microscope images and the major and minor axis were measured.


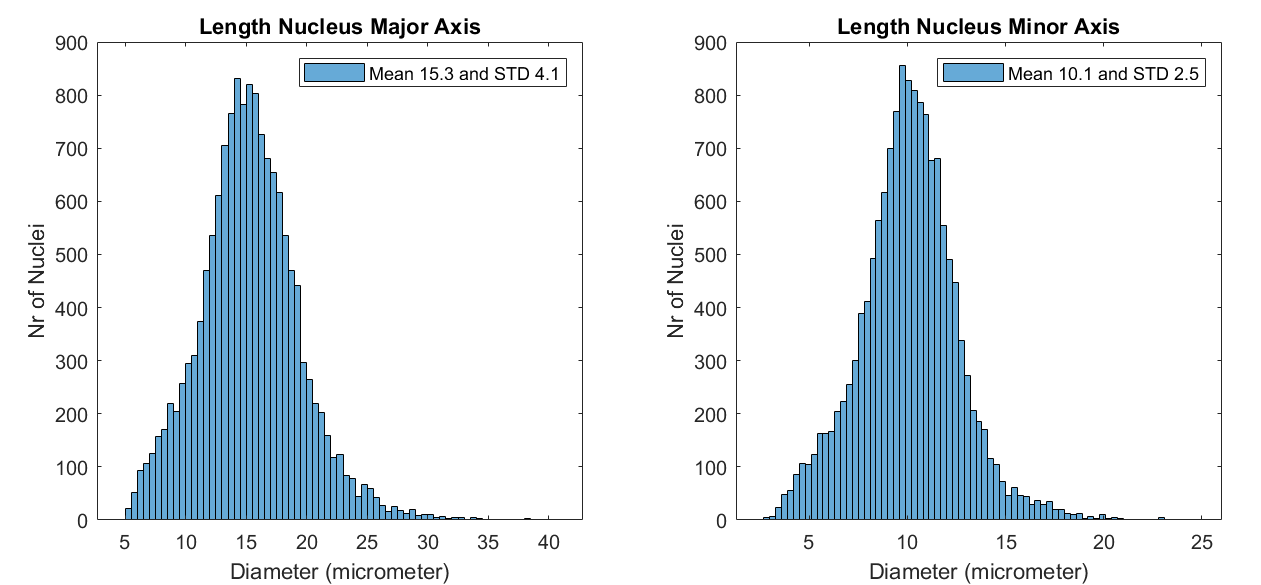


*Figure S4 Measured major (a) and minor (b) axis length of DAPI stained PC3 cell nuclei after image segmentation.*

*
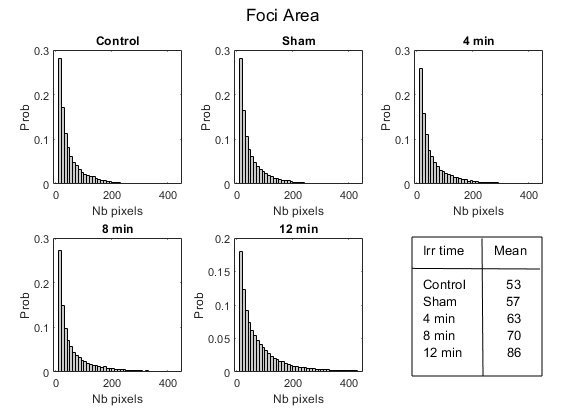
*

*Figure S5 Measured foci area (i.e., number of pixels) in DAPI stained PC3 cells in (a) control, (b) sham irradiated and cell irradiated 4 minutes (c), 8 minutes (d) and 12 minutes (e).*


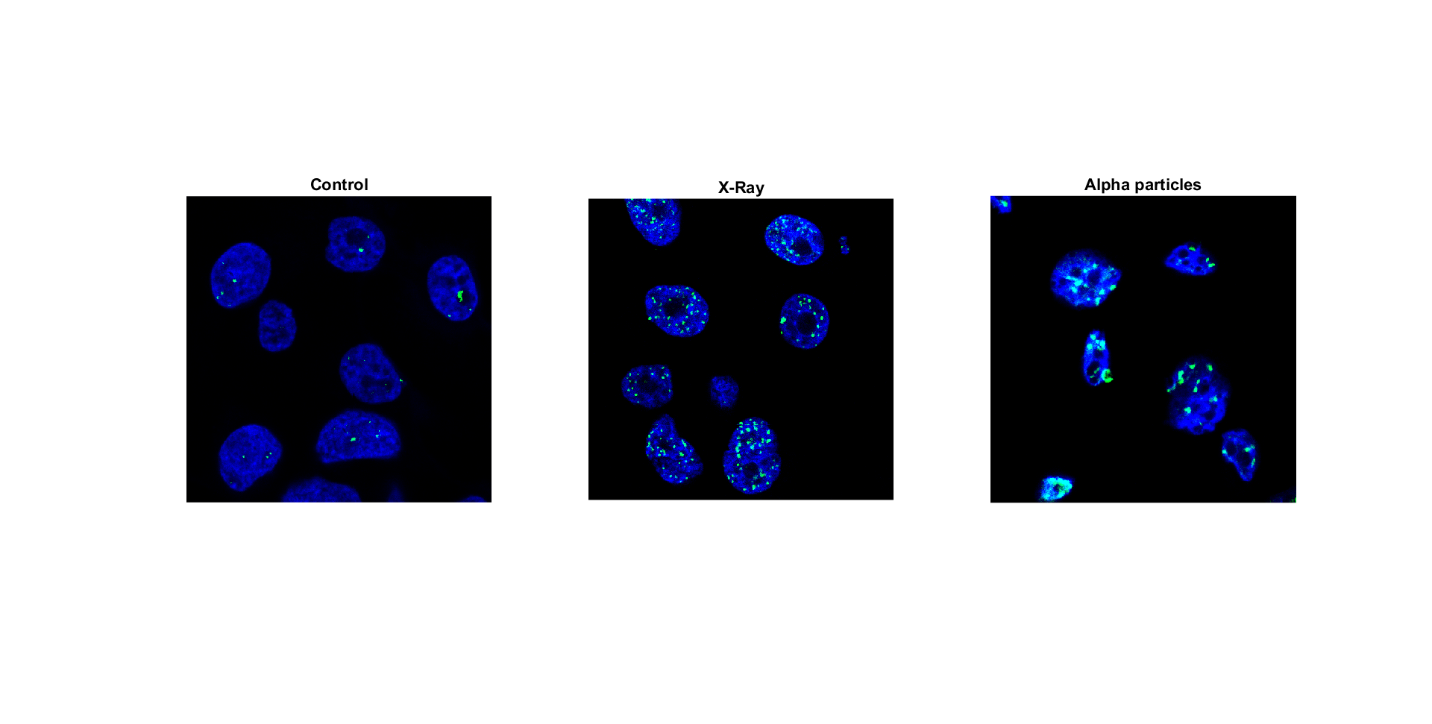


*Figure S6 Comparison of γ-H2AX foci (green spots) in DAPI stained PC3 cell nuclei (blue) in control (a), x-ray irradiated (b) and alpha particle irradiated (c).*


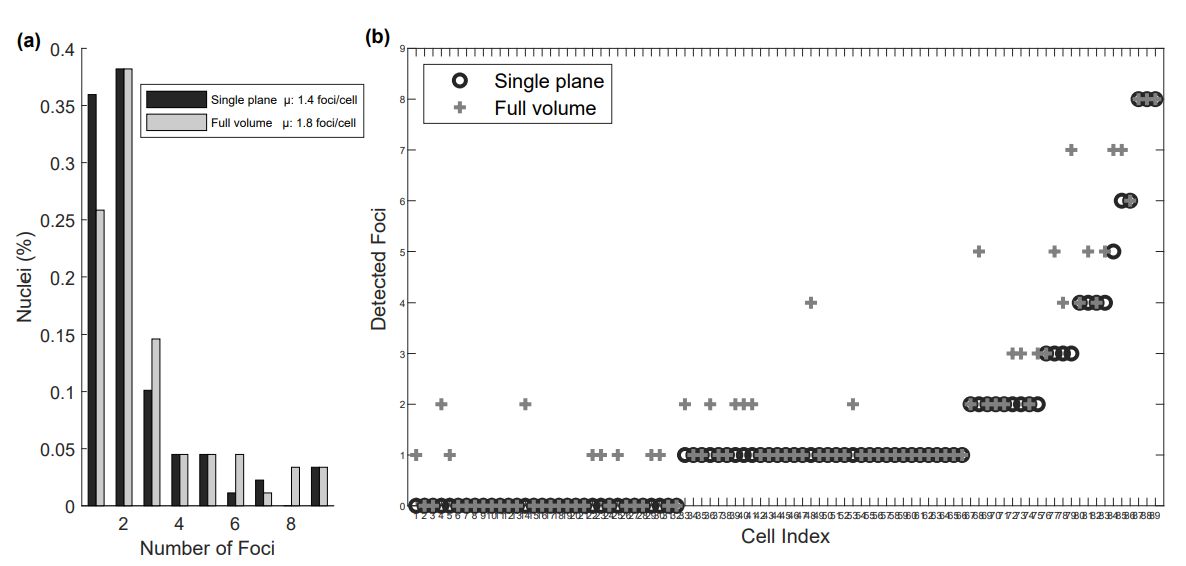


*Figure S7 The detected foci in 89 confocally imaged cell nuclei when only considering a central plane in the z-stack compared to the full nucleus volume. The distribution of foci in the cell nuclei (a) and a cell-by-cell comparison of number of detected foci (b).*

*Table S1 Summarized properties of the detected RIF distribution, and the simulated RIF distributions from the elliptical cylinder phantom and the mesh volume phantom after adjusting for missed foci.*

| Irradiation Time | Data | Mean | Median | Mode | Variance | SD | CV |
| --- | --- | --- | --- | --- | --- | --- | --- |
| 4 min | **Detected RIF** | 1.0 | 1 | 0 | 2.0 | 1.4 | 0.7 |
|  | **Simulated Ellipse RIF** | 1.0 | 1 | 0 | 1.2 | 1.1 | 0.9 |
|  | **Simulated Mesh RIF** | 0.9 | 1 | 0 | 1.2 | 1.1 | 0.9 |
| 8 min | **Detected RIF** | 1.7 | 1 | 0 | 2.1 | 2.1 | 0.8 |
|  | **Simulated Ellipse RIF** | 1.9 | 2 | 1 | 1.6 | 1.6 | 1.2 |
|  | **Simulated Mesh RIF** | 1.9 | 2 | 1 | 1.6 | 1.6 | 1.2 |
| 12 min | **Detected RIF** | 2.9 | 3 | 2 | 4.3 | 2.1 | 1.4 |
|  | **Simulated Ellipse RIF** | 2.9 | 3 | 2 | 4.1 | 2.0 | 1.4 |
|  | **Simulated Mesh RIF** | 2.8 | 2 | 2 | 4.4 | 2.1 | 1.3 |
